# The Impact of COVID-19 Pandemic on Food Security and Food Diversity of Iranian Rural Households

**DOI:** 10.3389/fpubh.2022.862043

**Published:** 2022-03-31

**Authors:** Rezvan Ghanbari Movahed, Fatemeh Maleki Fard, Saeed Gholamrezai, Mohammad Reza Pakravan-Charvadeh

**Affiliations:** Department of Rural Development, Faculty of Agriculture, Lorestan University, Khorramabad, Iran

**Keywords:** food security, dietary diversity, rural households, COVID-19, Khorramabad

## Abstract

With the onset of the coronavirus crisis, disruption of the domestic food supply chain, loss of revenue, and payments that affect food production have led to severe tensions and food security risks in many developing countries. The rural communities are more at risk of food insecurity due to less access to healthcare and social inequality. Therefore, this study aimed to assess the impact of the COVID-19 pandemic on food security and food diversity of rural households. The sample included 375 household heads living in the rural areas of Khorramabad county, which was determined using a three-stage cluster sampling method. Data were collected using standard Household Food Insecurity Access Scale (HFIAS) and Household Dietary Diversity Score (HDDS) questionnaires. The results showed that the food security situation of rural households has deteriorated, and consumption of some food groups changed during the COVID-19 pandemic. The results of the multinomial regression model showed that gender, level of education, monthly income, number of employed members, nutrition knowledge, employment status, livestock ownership, and access to credit were significantly associated with the food security of households during the COVID-19 pandemic. The household head's gender, level of education, monthly income, nutrition knowledge, employment status, livestock ownership, and access to credit were significantly associated with dietary diversity during the COVID-19 pandemic. Based on the findings, providing emergency food assistance and cash payments to food-insecure households can reduce the risk of food insecurity in rural households. It is suggested that government policies focus on identifying vulnerable households in rural areas, especially female-headed households, low-income households, and households without a wage income.

## Introduction

The COVID-19 has formed a pandemic that is very different from previous pandemics and covers almost all countries around the world, especially the major economies. In addition to the negative effects on health, the COVID-19 crisis has put both people's lives and livelihoods at risk (1, 2). COVID-19 threatens the years of progress in health care, hunger, poverty, and education. Since the great depression, the world has been facing the worst recession. Real per capita gross domestic product (GDP) fell by 3.3 percent in 2020, and it is estimated that the economic instability created by the global epidemic has led to the loss of 114 million jobs worldwide (3). COVID-19 also threatens access to food mainly through the loss of income and assets, which impairs the ability to buy food. The effects of COVID-19 have led to a sharp and widespread increase in global food insecurity, which affects vulnerable families in nearly all countries, and are expected to endure through 2022 and perhaps beyond. The poorest households spend about 70 percent of their income on food and have limited access to financial markets, which makes food security vulnerable to income shocks (4–6). More than half of the world's undernourished are found in Asia (418 million), and a significant portion of these people live in rural areas (7). Thus, food security in rural areas has become a significant issue in global decision-making and is considered a major challenge for national policies and public concerns (8, 9). More than two billion small producers, farmworkers, rural workers, and their families, who represent a large section of the population affected by food insecurity, are affected by the economic shock caused by and their incomes are at risk (10). In developing countries, most of the livelihoods of rural households come from the agricultural sector (11). Hence, rural households are the most affected by poverty and vulnerability and face economic, financial, and social risks (12). According to the clinical studies, the lack of micronutrients is one of Iran's main health and nutritional problems, especially in rural areas, so the rural community is facing food insecurity problems, especially among women and children (13). Results of the previous studies have shown that one-third (32.4%) of all rural households in Iran were faced with food insecurity, which is determined based on per capita calories consumed. They have lower socioeconomic status, experience more food shortages than others, have less chance of buying the healthy and nutritious foods offered, and generally consume fewer types of fruits and vegetables. Also, 50% of rural households in Iran are deficient in iron, calcium, iodine, and a variety of vitamins (14–16). Therefore, they are at risk of a food crisis until measures are taken to protect this vulnerable group. Global and national interventions are necessary to reduce the impact of the COVID-19 pandemic across the food system. Measures taken to maintain and reorganize food supply chains should be complemented by specific solutions using locally available resources and goods (17).

Due to the importance of the different impacts of COVID-19 on food security and nutrient status, some scholars endeavored to assess and calculate these effects. Nechifor et al. (18) claimed 1.3% of the participated households still fall below calorie intake thresholds in sub-Saharan Africa, especially in rural areas. Results also showed that food security remains vulnerable to the growth of the pandemic abroad in Kenya. Yazdanpanah et al. (19) reported that the food security of rural households in southern Iran reduced during the COVID-19 pandemic. The regression analysis results also showed that financial, psychological, physical, and human assets affect the food security of rural households under COVID-19. Pakravan et al. (20) demonstrated that the food security status of Iranian households has improved at the first stages of COVID-19 pandemic disease. In fact, they believed that the impacts of the COVID-19 should be analyzed in different periods that include short-term and long-term time. Ceballos et al. (21) assessed the short-term effects of COVID-19 on the food security of rural households in Guatemala. They found that during COVID-19, food security and food diversity among rural households decreased due to rising prices, decreasing incomes, and reduced access to food in local markets. Egwue et al. (22) reported that most of Nigeria's rural households were food insecure during COVID-19. The results also showed that marital status, education level, cooperative membership, and annual income of heads of households positively affected food security. In contrast, the household head's age and household size negatively affected the food security status of rural households. Cardarelli et al. (23) contended that although the COVID-19 pandemic initially reduced access to food and disrupted food supply, in the long run, rural households' access to food has increased through federal aid. Ouoba and Sawadogo (24) reported that the COVID-19 pandemic disease had reduced households' incomes in Burkina Faso by increasing their likelihood of entering poverty. They showed that the households could adjust to the shock (COVID-19) during a long-term period. Rahman et al. (25) contended that during COVID-19, the incomes of certain groups of people declined, which may have contributed to the growth of the poverty rate. Also, quarantine, movement and social restrictions, agro-food systems, supply–value chains, and market levels were affected. Also, the overall state of food consumption was affected by the COVID-19 pandemic throughout the country and affects all parts of the population.

This paper assesses the impact of the COVID-19 pandemic on food security and food diversity of rural households in the Khorramabad township. Whereas, most of the previous studies have tried to assess food security in urban areas, in this study, we tried to focus on local levels, where people are more vulnerable and affected by the hazard. The findings of this study can help better understand the rural population's needs during crises such as the COVID-19 epidemic. This can help better plan and take preventive measures for such populations after understanding their needs. In addition, it can help deprived people to have a minimum level of preparedness and food security in such a crisis in the future. Therefore, this study tries to address the following objectives:

- To determine the food security status of rural households before and during COVID-19 in Khorramabad township
- To assess food diversity status of rural households before and during COVID-19 in Khorramabad township
- Identify the factors associated with food security and food diversity of rural households before and during COVID-19 in Khorramabad township.

## Methodology

### The Study Area

**Figure 1:**
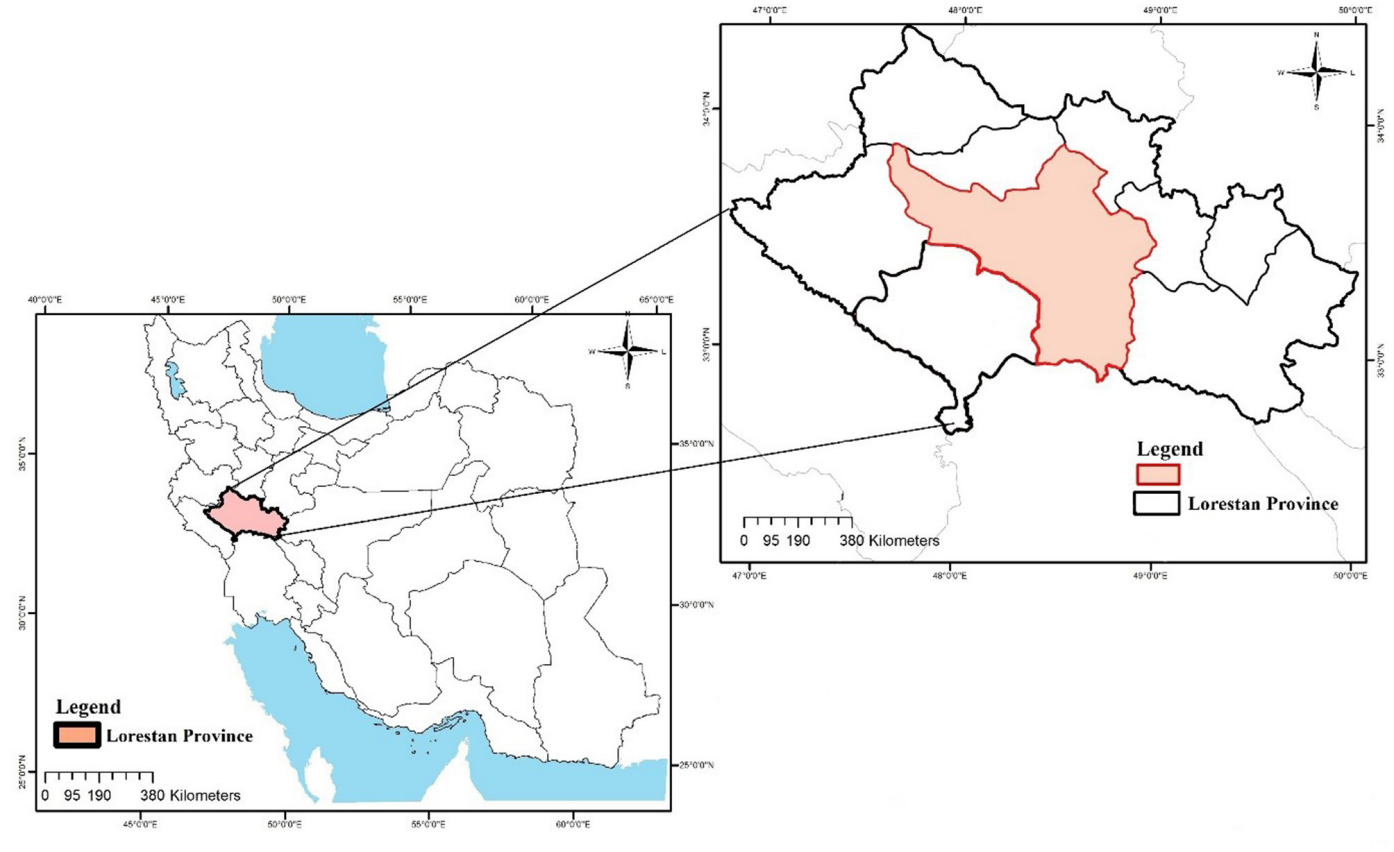
Study area.

This cross-sectional research was conducted between July and August 2021 in rural areas in Khorramabad county in Lorestan Province. Khorramabad county has a total population of 506,741 people, and 124,417 of them live in rural areas (26). The county lies between the latitudes 48°16′ N to 48°24 N and longitudes 33°26′ E to 33°34′ E covering an area of 6,450 km^2^ (Figure 1).

### Data Collection

The study population included 375 household heads living in rural areas of Khorramabad county. The correlational research model was used in the research. Cochran's formula was used to calculate the finite sample size as follows:


(1) $$\begin{array}{r} n=\frac{\frac{t^{2}pq}{d^{2}}}{1+\frac{1}{N}\left(\frac{t^{2}pq}{d^{2}}-1\right)} \end{array}$$

In this formula, *n* is the sample size, *t* equals 95% (the error rate equals 1.96), *N* is the population size, *p* represents the presence of trait and equals 50%, *q* is the lack of trait. It equals 50%, and *d* stands for the probability of error and equals 0.05. After the values were put in the Cochran's formula, the sample size was estimated to be 375 individuals. The questionnaire used in this study consists of three parts. The first part includes questions related to personal, economic, and social characteristics that were gathered from previous studies, and the second part includes the International Standard Household Food Insecurity Access Scale (HFIAS) (27), which has been validated by Salarkia et al. in Iran (28). The third part measures household food access using the Household Dietary Diversity Scale (HDDS).

### Data Analysis

The collected data were analyzed using SPSS version 24 software. This study used frequency, mean and standard deviation for descriptive statistics, Pearson's, a paired sample *t*-test, chi-square, multinomial regression, and chi-square tests were used for inferential statistics.

### Measuring Food Security

#### The Household Food Insecurity Access Scale

Different dimensions of food security make it possible to use different indicators and scales to assess food security; hence, no single indicator can simultaneously evaluate the four dimensions of food security (29). The various methods and tools used in food security assessment indicate its operational concept's complexity (30). The Household Food Insecurity Access Scale (HFIAS) is a recently developed approach that measures households' perception of their access to food and does not include food consumption or nutritional outcomes (31). Household food insecurity was determined using HFIAS on a nine-item scale over a reminder period of the past 4 weeks (30 days) (27). For each positive answer, the person used the four-point scale (never, rarely, sometimes, often) to provide additional information for the frequency, and their total score is from 0 to 27. A high HFIAS showed a high level of food insecurity for households (32). The prevalence of household food insecurity was classified into four levels: food secure, mild, moderate, and severe food insecurity. The percentage of each category was analyzed using the indicator Guide version 3 (27).

### Measuring Dietary Diversity

#### The Household Dietary Diversity Scale

The dietary diversity is a qualitative scale to measure household access to various foods and reflects the nutrient adequacy in the diet of all household members for productive life (33). The dietary assessment questionnaire was according to the FAO instructions for measuring dietary diversity of households (34). The HDDS is described as the number of food groups a household consumes in the specified period (usually based on the previous 24 h). The following 12 food groups are used to measure the HDDS indicator: white tubers and roots, cereals, legumes, nuts and seeds, vegetables, fruits, meat, eggs, milk and milk products, fish and other seafood, oils and fats, spices, sweets, beverages, and condiments. Dietary diversity was summarized to create dichotomous occurrence variables for each food group and indicators associated with each group (35). The HDDS variable was measured by adding the count of food groups consumed by the household, and scores ranged from 0 to 12 (36). A lower score indicates lower household dietary diversity (37).

## Results

### Food Security Status

**Figure 2:**
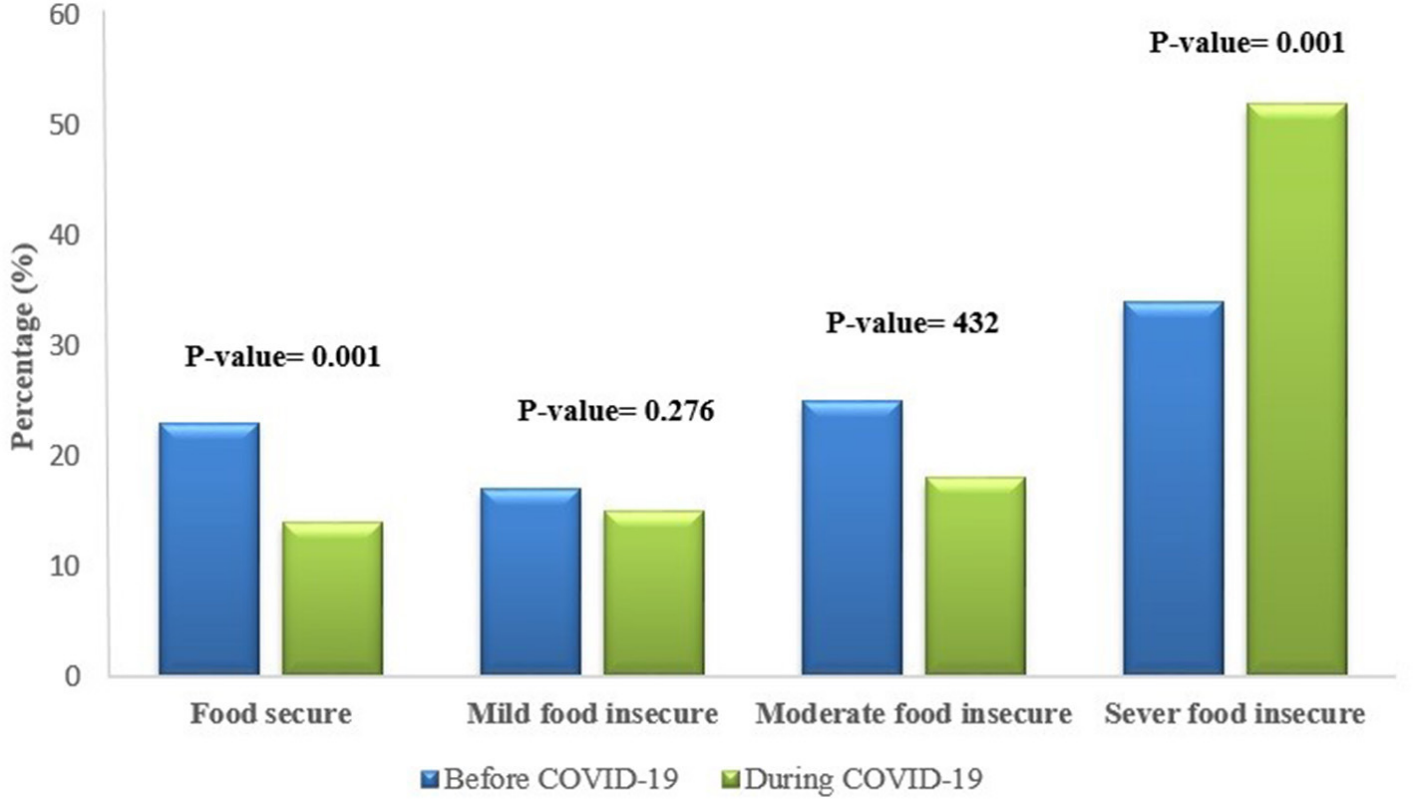
Food security status of rural households before and during COVID-19.

Table 1 shows the food security status of rural households before and during the COVID-19 pandemic. The results showed that there is a significant difference between food security items before and during the COVID-19. The worry about not having enough food has increased among rural households, and eating a variety of foods has limited. According to the mean of questions 8 and 9, rural households' hunger level was higher in the COVID-19 condition than before. Figure 2 shows that there was a significant difference between the food security status of rural households before and during the pandemic. About 34.5% of rural households were severe food insecurity before the COVID-19 pandemic, which increased to 52.5% during the COVID-19 pandemic. The percent of rural households that were food secure decreased from 21 to 14% during COVID-19. There was no significant difference between mild and moderate food insecurity during the COVID-19 pandemic compared to before. Also, the results showed that about 52.5% of rural households need urgent assistance to struggle with inappropriate food insecurity conditions.

**Table 1:** Results of the HFIAS questionnaire of the studied households.

**Question items**	**Never**		**Rarely**		**Sometimes**		**Often**		**Mean**
	***N***	**%**	***N***	**%**	***N***	**%**	***N***	**%**	
Worry about food	59	15.8	81	21.7	101	27.1	134	35.4	1.23
Unable to eat preferred foods	70	18.6	96	25.7	106	28.2	103	27.5	0.99
Eat a limited variety of foods	72	19.3	91	24.4	114	30.5	98	25.8	0.96
Eat foods that you did not want to eat	117	31.2	83	22.1	99	26.5	76	20.2	0.94
Eat a smaller meal	133	35.4	88	23.6	84	22.4	70	18.6	0.81
Eat fewer meals in day	138	36.7	101	27.1	75	19.9	61	16.3	0.84
No food to eat of any kind in the household	199	53.1	89	23.5	57	15.3	30	8.1	0.53
Go to sleep at night hungry	230	61.3	86	23.1	35	9.3	24	6.3	0.47
Go a whole day and night without eating	236	63	96	25.6	26	6.9	17	4.5	0.44

### Socioeconomic and Demographic Characteristics of Participants

Table 2 shows the scale for measuring all variables used in this study. Further, describing and explaining the variables, we present them in two categories of discrete and continuous variables as follows.

**Table 2:** Description of the variables studied in the research.

**Variables**	**Measurement**	**Description**
**Dependent variables**
Food security status	0–27	Household food security in last 4 weeks (food security = 0, sever food insecurity = 27)
Dietary diversity status	0–12	Number of food groups consumed by a household in last 24 h
**Independent variables**
Age	Number	Age of household heads (number of years)
Education level	1–4	household heads' education level (1 = no formal education 2 = primary education 3 = secondary education 4 = tertiary education)
Household size	Number	Total members in the household (number of person)
Household type	1–2	The household type included (1 = nuclear: father, mother, unmarried family members, 2 = extended: father, mother, married family members, grandsons, grandpa, grandma = 2)
Household head's gender	1–2	Gender of household's members head (1 = male 2 = female)
Household head's employment status	1–3	Household head's occupation (1 = employed 2 = unemployed 3 = seasonal)
Children under 18 years	Number	Total children under 18 years in the household (number of people)
Being under the coverage of a supporting center	0–1	Household being under the coverage of a supporting center, for example financial aid organizations, NGOs (if supporting = 1, otherwise = 0)
Access to credit	0–1	Access of households to credit (credit received = 1, otherwise = 0)
Household head's monthly income	1–4	Household head's income group based on Rial, Iran's currency (1 = 0–14,000,000, 2 = 14,000,010–28000000, 3 = 280,000,010–42,000,000, 4 = 420,000,010-56,000,000)
Household employed members	Number	Total employed members in the household (number of person)
Personal saving	0–1	Whether a Household head have a personal saving in a bank (has personal saving = 1, otherwise = 0)
Participate in home loans	0–1	Whether Household head has participate in home loans (has participate = 1, otherwise = 0)
Land personal's ownership	0–1	Household head's land personal's ownership (has land personal's ownership = 1, otherwise = 0)
Farm size	Number	Farm size of a household (number of hectares)
Livestock ownership	Number	Livestock ownership of household (number of units)
Distance to market	Number	Distance to market of household (number of a kilometer)
Nutrition knowledge	1–5	Household head's nutrition knowledge) 1 = very low 2 = low 3 = medium 4 = high 5 = very high)

### Descriptive Statistics of Continuous Variables

Table 3 shows the scale for measuring all variables used in this study. Further, describing and explaining the variables, we present them in two categories of discrete and continuous variables as follows.

**Table 3:** Descriptive statistics of continuous variables.

**Variable**	**Mean**	**minimum**	**Maximum**	**SD**
Age	47.25	22	75	9.46
Household size	4.20	2	13	1.16
Household employed members	0.42	0	4	0.77
Children under 18 years	1.01	0	4	1.13
Farm size	4.23	0	55	10.68
livestock ownership	5.89	0	110	12.55
Distance to market	26.74	2	65	10.45

### Descriptive Statistics of Discrete Variables

Descriptive analysis of the categorical variables is described in Table 4. About 89% of the household head were male, whereas 41% of them had secondary education. More than 90 percent of households are nuclear. The findings showed that about 96% of the respondent households did not participate in nutrition training classes. About 81% of the respondent households were not under a supporting center's coverage, whereas only 30% of household heads had a permanent job. Almost 65% of households were in the first income group in terms of monthly income, and 92% of households had no personal savings. About 43% of the households had access to credit, and 49% participated in home loans.

**Table 4:** Descriptive statistics of discrete variables.

**Variable**	**Category**	***N***	**%**	**Mode**
Household type	Nuclear	345	92	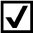
	Extended	30	8	
Household head's gender	Male	334	89.2	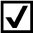
	Female	41	10.8	
Household head's level of education	No formal education	62	16.7	
	Primary education	132	35.2	
	Secondary education	155	41.3	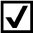
	Tertiary education	26	6.8	
Household head's employment status	Employed	114	30.5	
	Unemployed	156	41.6	
	Seasonal	105	27.9	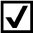
Participate in family nutrition training class	Yes	15	3.8	
	No	360	96.2	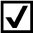
Being under the coverage of a supporting center	Yes	71	18.8	
	No	304	81.2	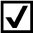
Access to credit	Yes	163	43.6	
	No	212	56.4	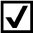
Household head's monthly income	Group 1	243	64.9	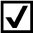
	Group 2	108	28.8	
	Group 3	19	5.1	
	Group 4	5	1.3	
Personal saving	Yes	29	7.6	
	No	346	92.4	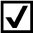
Participate in home loans	Yes	186	49.6	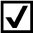
	No	189	50.4	
Land personal's ownership	Yes	16	4.3	
	No	359	95.7	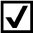

### Dietary Diversity Status

Table 5 shows the households' dietary diversity status before and during the COVID-19 pandemic. The households' dietary diversity score showed that the total average of food groups of the studied households is 7.75. Consumption of meat, fruits, and eggs among households has decreased during the COVID-19 pandemic, despite the increase in consumption of cereals, legumes, sweets, spices, condiments, and beverages. Also, the HDDS score of households before COVID-19 (8.06) is higher than during COVID-19 (7.11). The highest level of food group consumption is related to cereals with an average of (1.23), and the lowest belongs to fish consumption and other seafood (0.10).

**Table 5:** Food groups status rural households.

**Food groups**	**Household heads**									***p*-value**
				**Before COVID-19**			**During COVID-19**			
	***N***	**%**	**mean**	***N***	**%**	**mean**	***N***	**%**	**mean**	
Cereals	367	97.8	0.98	363	96.8	0.97	374	99.8	1.23	0.002
White tubers and roots	165	44.1	0.44	144	38.5	0.39	156	41.7	0.42	0.182
Vegetables	346	92.2	0.90	353	94.2	0.94	346	92.3	0.88	0.089
Fruits	229	61.2	0.65	264	70.5	0.71	185	49.5	0.49	0.005
Meat	204	54.4	0.46	210	56.1	0.58	181	48.3	0.46	0.001
Eggs	207	55.3	0.57	237	63.2	0.62	193	51.4	0.50	0.034
Fish and other seafood	35	9.4	0.09	32	8.5	0.08	36	9.7	0.10	0.537
Legumes, nuts and seeds	182	48.5	0.49	181	48.3	0.49	223	59.6	0.61	0.003
Milk and milk products	272	72.5	0.74	272	72.6	0.72	278	74.1	0.73	0.588
Oils and fats	309	82.5	0.82	310	82.7	0.87	355	94.8	1.45	0.216
Sweets	294	78.3	0.77	271	72.3	0.76	301	80.5	0.85	0.024
Spices, condiments, and beverages	300	80.2	0.81	294	78.5	0.79	340	90.6	0.97	0.035
Total mean food groups			7.75			8.06			7.11	
Min			2							
Max			12							

**Figure 3:**
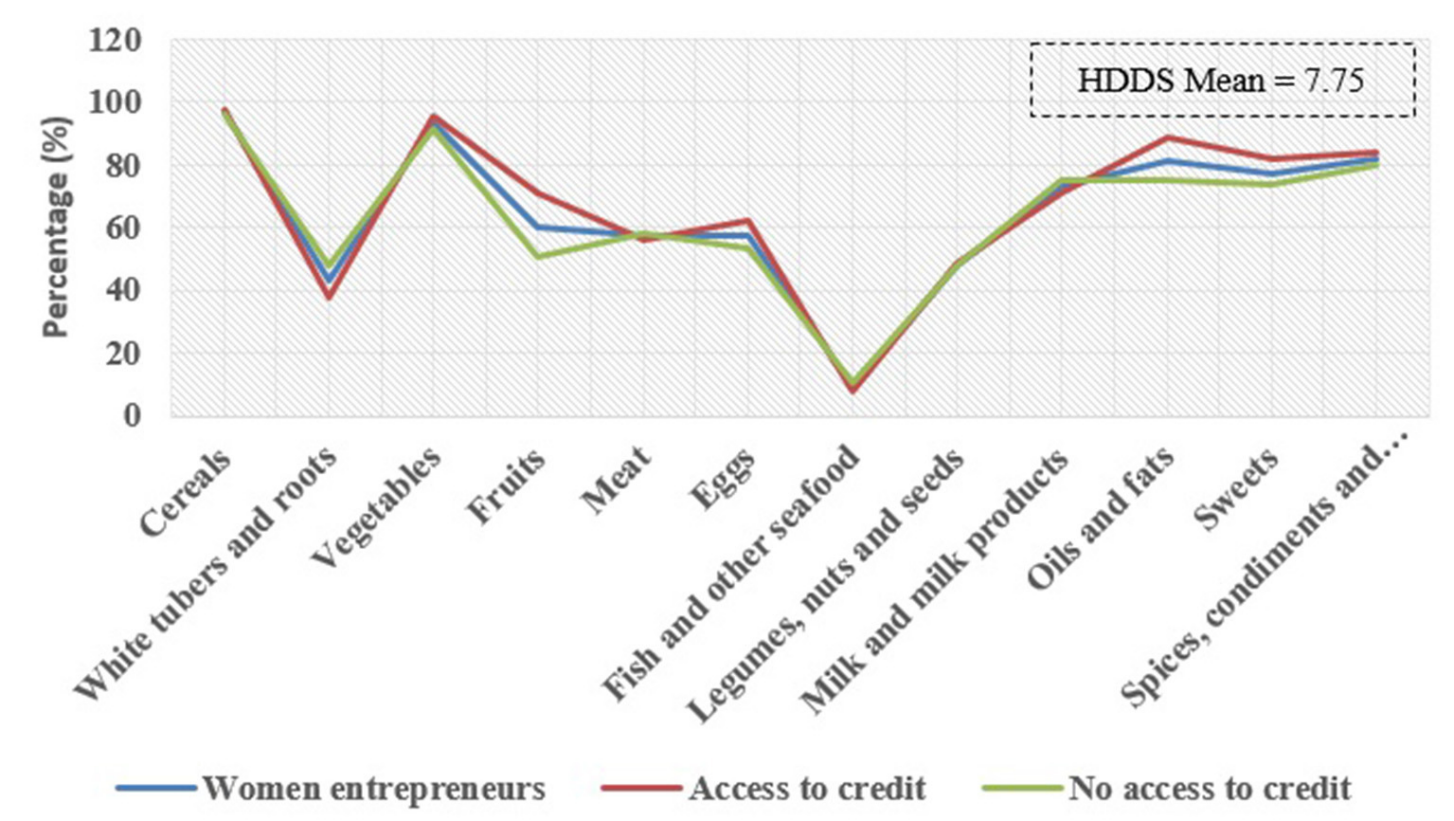
Dietary diversity status of rural households before and after the COVID-19 pandemic.

Figure 3 shows that rural households before COVID-19 have a better situation compared to the during COVID-19 time in terms of consumption of food groups (meat, eggs, and fruits). Also, one of the most important outputs of this chart is the low consumption of fish and seafood before and during the COVID-19 pandemic.

### Multinomial Regression Model Results

A multinomial regression model was used to estimate the association of socioeconomic factors with the level of food security of rural households. Table 6 shows the results of this model for food security status before and during the COVID-19 pandemic. Before running the multinomial regression model, the variance inflation factor (VIF) index was used to assess the multicollinearity of independent variables. Since the VIF values for each variable were <5, there is no multilinear concern between the variables. Also, based on the likelihood ratio test (*p* = 0.000), the model has strong explanatory power. This model showed that the variables of the household head's gender, level of education, monthly income, number of employed members, personal saving, size of household, and nutrition knowledge have a significant association with the level of food security of rural households before COVID-19 pandemic. The household head's gender, level of education, household head's monthly income, number of employed members, nutrition knowledge, household head's employment status, livestock ownership, and access to credit are directly associated with the food security of rural households during the COVID-19 pandemic.

**Table 6:** Factors associated with food security before and during COVID-19 using multinomial regression.

**Variables**	**VIF**	**Before COVID-19**			**During COVID-19**		
		**Coefficient**	**Odd**	***p*-value**	**Coefficient**	**Odd**	***p*-value**
Intercept		−4.080		0.066	−2.433		0.329
Household head's gender	1.141	0.988	1.022	0.001	0.059	1.067	0.001
Size of household	1.355	−0.322	1.044	0.032	−0.87	1.034	0.121
Household type	1.231	0.165	1.028	0.765	0.345	1.008	0.532
Participate in home loans	2.187	0.561	1.076	0.065	0.230	0.801	0.087
Children under 18 years	1.206	−0.078	1.027	0.241	−0.088	1.132	0.212
Livestock ownership	2.144	0.546	2.998	0.079	0.126	1.750	0.010
Access to credit	1.258	1.008	1.562	0.023	0.657	0.421	0.003
Farm size	1.342	0.109	1.223	0.058	0.109	0.615	0.234
Distance to market	1.203	−0.018	0.532	0.368	−0.022	3.854	0.896
Education level	1.175	1.021	1.404	0.841	0.309	2.312	0.001
Number of employed members	1.288	0.980	0.512	0.014	0.280	0.736	0.014
Nutrition knowledge	1.142	1.143	1.205	0.005	0.463	1.005	0.004
Household head's monthly income	1.344	1.015	1.053	0.003	0.854	1.133	0.000
Personal saving	1.432	0.845	1.023	0.030	0.232	1.021	0.843
Being under the coverage of a supporting center	2.312	0.360	1.070	0.621	0.654	0.698	0.027
Household head's employment status	1.432	1.01	1.125	0.410	0.850	1.243	0.009

Table 7 shows the association of socioeconomic factors with the level of dietary diversity of rural households. Before estimating the model, the collinearity between the variables was checked using VIF. The value of the VIF coefficient for all variables in the model was less than five. The results of the multinomial regression model showed that the variables of the household head's gender, household head's level of education, household head's monthly income, household size, and nutrition knowledge were directly associated with the dietary diversity score of rural households before the COVID-19 pandemic. Also, the results showed that the household head's gender, level of education, monthly income, nutrition knowledge, employment status, livestock ownership, and access to credit have a significant association with the level of food security of rural households during the COVID-19 pandemic.

**Table 7:** Factors associated with dietary diversity before and during COVID-19 using multinomial regression.

**Variables**	**VIF**	**Before COVID-19**			**During COVID-19**		
		**Coefficient**	**Odd**	***P*-value**	**Coefficient**	**Odd**	***P*-value**
Intercept		−4.080		0.066	−2.433		0.329
Household head's gender	1.243	−0.134	1.324	0.001	−3.834	1.211	0.001
Size of household	1.243	0.272	1.211	0.032	0.079	1.068	0.121
Household type	1.367	0.012	1.030	0.765	0.034	1.213	0.532
Participate in home loans	2.578	0.341	1.405	0.065	0.018	1.435	0.087
Children under 18 years	1.421	−0.046	1.612	0.241	−0.250	1.654	0.212
livestock ownership	2.187	0.036	2.017	0.079	0.145	1.0576	0.010
Access to credit	1.345	1.045	0.657	0.023	0.743	0.324	0.003
Farm size	1.421	0.054	0.523	0.058	1.432	0.089	0.234
Distance to market	1.176	−0.015	0.324	0.368	−0.209	0.456	0.896
Education level	1.230	1.085	2.401	0.841	1.765	1.126	0.001
Number of employed members	1.324	0.987	0.531	0.414	0.467	0.123	0.314
Nutrition knowledge	1.423	1.237	1.006	0.005	0.798	2.056	0.004
Household head's monthly income	1.211	0.135	1.087	0.003	3.309	1.080	0.000
Personal saving	1.542	−0.819	0.765	0.130	0.099	1.012	0.843
Being under the coverage of a supporting center	2.165	0.543	1.098	0.621	0.065	1.126	0.027
Household head's employment status	1.219	−0.788	1.376	0.410	1.002	1.567	0.009

## Discussion

The main aim of this study was to examine the impact of the COVID-19 pandemic on food security and food diversity of rural households. In the first step, the food security situation of rural households was assessed. The results showed that 14% of rural households have food security, and 52.5% need urgent assistance to struggle with inappropriate food insecurity conditions. This finding is consistent with the results of other studies (19, 25, 38). In developing countries, most of the livelihoods of rural households come from the agricultural sector (39, 40). Hence, rural households are most affected by poverty and vulnerability and face economic, financial, and social risks (41). COVID-19 disrupts various stages of the food supply chain and simultaneously affects farm production, food processing, access to inputs, transportation, logistics, and consumer's demand. Decreased exports of food and agricultural products due to the closure of borders and health quarantines are other consequences that can lead to the accumulation and cheaper products. Another problem is labor shortages due to the fear of transmitting the virus, which ultimately leads to the waste of the product (42–44). Thus, the income of many rural households has been reduced due to restrictions and quarantine measures and business closures (45). Access to valuable foods has been reduced and replaced by high-calorie, low-value foods, which have led to obesity along with cell starvation, increasingly putting rural people at risk for coronavirus disease and other diseases (46, 47).

Our results on the dietary diversity of rural households showed that the consumption of some food groups changed during the COVID-19 pandemic. Consumption of meat, fruits, and eggs among households has decreased during the COVID-19 pandemic. This finding aligns with several recent studies that reported rapid changes in diets and food consumption habits during the COVID-19 pandemic (46, 48, 49). During the COVID outbreak, due to the increase in the price of animal protein sources, the access of low-income decile groups to these items has decreased (50). Even the results of recent studies by the Ministry of Health and medical education in Iran have also shown that due to the increase in prices by up to 35%, the consumption of some food items including red meat, chicken, milk, dairy products, and fruits has decreased in Iranian households that the continuation of this issue can lead to food insecurity and malnutrition. Therefore, serious government oversight is essential to control food prices; protein sources should be available to people at a reasonable price, especially vulnerable groups. During the COVID-19 pandemic, it is very important to have a proper function of the immune system following proper nutrition, which the body fights against the disease in case of COVID-19 infection, and even after getting a COVID-19 vaccine, the vaccine response is very much related to the immune system function (51).

Also, the results showed that the consumption of cereals and legumes among rural households increased during COVID-19. The results of the previous studies also confirm this finding (52, 53). Rural households prefer to use legumes as a cheaper source of protein in the diet. Legumes are a good alternative to meat, milk, and eggs for a lower price. However, that does not mean that people can stay healthy, get the protein, and provide the protein their bodies need. Proper nutrition plays an important role in strengthening the immune system. Basically, in people whose immune systems are weakened due to poor nutrition, the body's resistance to the virus is usually lower, and the severity of the disease and the recovery period may even increase (46). Also, increasing grain consumption among rural households can cause them to gain weight during the COVID-19 pandemic. This finding is consistent with the previous studies (52, 54) that have reported increased cereal intake during the COVID-19 epidemic, which leads to more meals per day and weight gain.

We found that the consumption of sweets and sugars among rural households increased during the COVID-19 pandemic, which can increase the risk of COVID-19 infection by stimulating the immune system. In line with this, the results of a study in Norway showed that due to the psychological stress caused by COVID-19, the consumption of high-sugar foods has increased among households (55). Some people struggle with sugar and unhealthy foods as a way to replace stress (56). Eating sugary and highly caloric foods can also affect feelings such as loneliness during social isolation (57, 58).

The results showed that the head's gender was directly associated with food security and dietary diversity during the COVID-19 pandemic. Other scholars have also reported a female-headed households were more likely prone to food insecurity (59). The female-headed households faced challenges such as lower-income, limited access to capital, land ownership, market, and new technologies (60, 61). Also, most female-headed households in rural areas lack access to information and natural resources, which significantly affects increasing food insecurity (62). They tend to be in more precarious positions, earn lower-income, and epidemic may also expose them to higher levels of stress and violence (63–65). Therefore, gender-sensitive design and implementation of social protection interventions are crucial to ensure that rural women can participate in and equally benefit from these interventions.

Household head's employment status was positively and significantly associated with the food security of rural households. In other words, if the household head has a full-time job, less likely to experience a complete decline in income during the COVID-19 pandemic. Previous studies have confirmed the household head's employment status as a determinant of household's food security studies (38, 66, 67). Households with the unemployed head of households having either no fixed income or no income at all are more likely to have food insecurity (68). The purchasing power of these households is reduced due to a lack of sufficient income, so they cannot have sufficient access to food.

Household heads' monthly income was significantly related to the food security of rural households. Household heads put the food security situation of the family in a favorable position through the income from the income-generating activities. Some studies have shown a direct relationship between the income of household heads (in the agricultural and nonagricultural sectors) and food security (59, 69–71). High-income household heads are more likely to use food-based coping strategies during the COVID-19 epidemic to improve household nutrition. Therefore, their income can have a significant impact on the economic accessibility of the household. Higher-income households have greater food security because they have more choice in buying their household food (20). As income increases, household purchasing power increases, and they can prepare more food to meet their nutritional needs.

The education level of household heads was directly related to household food security. The higher education level of household heads likely provides more opportunities to find an appropriate job, thus enabling them to earn enough income to meet different nutritional needs (72). In rural areas, education influences food security through access to information on healthy nutrition in COVID-19 pandemic and quarantine conditions. Educated household heads are more likely to have more nutritional knowledge and know the importance of having all the nutrients and micronutrients in the family diet to maintain the immune system functioning to prevent and treat COVID-19 disease. They can also diversify household incomes, which increases the food supply of households (73).

The results showed that livestock ownership directly and significantly relates to household food security. Livestock ownership can reduce household food insecurity by increasing available disposable income that could be used to buy food and thus increase access to food. It also might directly increase the availability of livestock products for home consumption (74, 75).

The results showed that nutrition knowledge was significantly related to food security and diversity before and during the COVID-19 pandemic. This finding is in line with several recent studies that reported that increasing the level of nutritional knowledge of the head of the household can help perform appropriate nutritional behavior during the COVID-19 pandemic and improve the level of food diversity of households (20, 76). Adequate knowledge on the importance of a healthy diet during COVID-19 which can be optimal nutrition and dietary intake is the only sustainable way to survive in the current situation. A proper diet can ensure that the body can correctly defeat the virus (77). Basically, in people whose immune systems are weakened due to poor nutrition, the body's resistance to the virus is usually lower, and the severity of the disease and the recovery period may even increase (78).

## Limitations

In this study, several limitations need to be considered in future studies. First, because of the cross-sectional design, this study does not allow to draw causality, despite the use of retrospective data. Second, rural people are reluctant to provide their information due to a lack of trust in government agencies. However, we minimized their fears and built trust by clearly and openly communicating with them and providing transparency about using the data. Third, because the study was conducted during quarantine, access to research samples was difficult due to health protocols. Fourth, because many villagers did not wear masks and did not maintain social distance, communicating with them and distributing questionnaires was associated with problems such as fear of contracting the coronavirus. Finally, some heads of household did not remember the previous information, and we had to replace another household to complete the questionnaire.

## Conclusion

The results of our study showed that during the COVID-19 pandemic, food insecurity has increased among rural households, and they were concerned about not having enough food. Therefore, to prevent a food security crisis in rural communities, the government needs to develop support packages to support insecure households. Also, using the capacity of existing NGOs to identify and support food-insecure households in the current crisis can be very helpful. The government should provide sufficient funds to cover food-insecure rural households with free food baskets. The identification of these households will be possible using the capacity of the Iranian Welfare Database, which determines basket items and their necessities with the cooperation of nutritionists in the institutions in charge of nutrition in the country. Also, the results showed that the consumption of food groups was limited among rural households during the COVID-19. Due to quarantine and business closures, many rural households' incomes have been reduced to zero. Access to valuable foods has been reduced and replaced by high-calorie, low-value foods, which have led to obesity along with cell starvation, increasingly putting rural people at risk for coronavirus disease and other diseases. Therefore, the government can solve this problem with the right policies and allocating subsidies to the lower-income deciles so that the sources of protein and micronutrients enter the food basket of the people. Also, appropriate training and information by healthcare providers, the use of the capacity of health centers, health houses, and the social media in the field of healthy cooking training, and the use of nutritious and low-calorie foods and nutritional methods effective in improving the immune system among rural households can be effective in improving these conditions. Finally, government policies should focus on increasing the resilience of rural households to reduce their vulnerability to crises such as COVID-19. Therefore, the use of technology and cyberspace and changing the nature of some rural workshops and businesses should be on the agenda of the related institutions of rural policies to achieve a roadmap to strengthen businesses and employment of rural groups in the post-COVID-19 situation. Also, planting food crops and home gardening can be considered an option to increase the resilience of rural households in the medium term.

## Data Availability Statement

The datasets presented in this article are not readily available because due to the nature of this research, participants of this study did not agree for their data to be shared publicly, so supporting data is not available. Requests to access the datasets should be directed to ghanbari.re@lu.ac.ir.

## Author Contributions

RG: conceptualization, methodology, validation, resources, and data mining. FM: supervision, formal analysis, methodology, writing–original draft, and interpretation. SG: conceptualization, methodology, advisor, and investigation. MP-C: advisor, investigation, validation, questionnaire preparation, and writing, reviewing and editing. All authors contributed to the article and approved the submitted version.

## Conflict of Interest

The authors declare that the research was conducted in the absence of any commercial or financial relationships that could be construed as a potential conflict of interest.

## Publisher's Note

All claims expressed in this article are solely those of the authors and do not necessarily represent those of their affiliated organizations, or those of the publisher, the editors and the reviewers. Any product that may be evaluated in this article, or claim that may be made by its manufacturer, is not guaranteed or endorsed by the publisher.
